# 2210. Follow-up of Military Blood Donors Who Screen Positive for Syphilis

**DOI:** 10.1093/ofid/ofac492.1829

**Published:** 2022-12-15

**Authors:** Cara E Hojnoski, John Kieffer, Theresa Casey, Angela Osuna, Brian Casleton, Jason Okulicz, Joseph E Marcus

**Affiliations:** San Antonio Uniformed Services Health Education Consortium (Texas), Live Oak, Texas; 559th MDG/THLS Trainee Health, JBSA Lackland, Texas; JBSA Lackland Texas, JBSA Lackland, Texas; JBSA Lackland, JBSA Lackland, Texas; JBSA Lackland, JBSA Lackland, Texas; Brooke Army Medical Center, Texas, San Antonio, Texas; Infectious Disease - Brooke Army Medical Center, San Antonio, TX, San Antonio, Texas

## Abstract

**Background:**

Most data on the transmission and outcomes of syphilis occur in community sexually transmitted infection (STI) clinics and have shown increased risk for other STIs in patients who test positive. There is limited data on outcomes in other lower risk populations that undergo universal syphilis screening, such as blood donors. This study describes the initial evaluation, follow-up, and STI risk of Air Force military basic trainees who screen-positive for syphilis at blood donation.

**Methods:**

Blood donor data was gathered from the Armed Services Blood Bank Center-San Antonio for Air Force basic trainees who were screen-positive for syphilis between January 2014- September 2021. For each screen-positive case, six screen-negative controls were analyzed. Prospective donors were unable to donate if they reported male-to-male sexual encounters, HIV positive partners, or a bacterial STI in the prior year. Electronic medical records of cases and controls were examined for demographic information, STI history, and additional STI positivity within three years.

**Results:**

A total of 63,375 basic military trainees donated blood at the Armed Services Blood Bank Center-San Antonio during the study period, of which 23 (0.36 per 1,000 donors) screened positive for syphilis **(Table 1).** At time of screening, 3 (13%) trainees who screened positive for syphilis also tested positive for an additional STI. After initial treatment and screening, donors who screened positive for syphilis had a RR: 3.8 (95% CI 1.3-10.5, p=0.01) of developing an additional STI in the three years after blood donation compared to controls. Cases of N. gonorrhoeae **(Table 2)** in the 3-year period following blood donation were significantly higher in those who were syphilis screen-positive compared to those who were negative.

**Figure:**
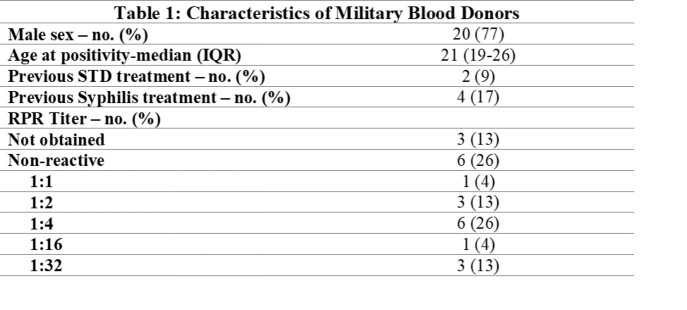

**Figure:**
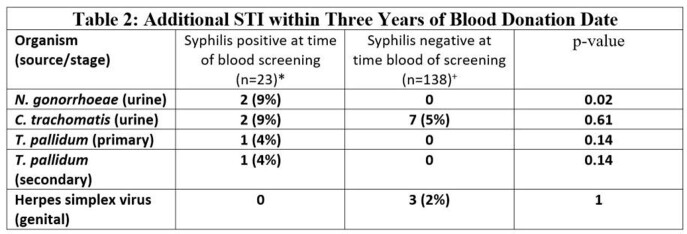

**Conclusion:**

There is limited data on follow-up of blood donors who screened positive for syphilis. This study found that in a low-risk population of military donors, those who screened positive for syphilis were at increased risk of future STIs compared to donors who were screen-negative. Syphilis screen-positive donors may be a potential target for future interventions to decrease the STIs burden in this population.

**Disclosures:**

**All Authors**: No reported disclosures.

